# Fowl typhoid live lyophilized vaccine applied at 3-month intervals protected layer chickens from *Salmonella gallinarum* infection and prevented cloacal shedding

**DOI:** 10.5455/javar.2022.i597

**Published:** 2022-06-28

**Authors:** Taslima Akter, Mohammed Nooruzzaman, Sheikh Mohammad Shariful Hoque Belal, Mustak Ahammed, ABM Jalal Uddin, Rokshana Parvin, Md. Abu Hadi Noor Ali Khan, Md. Ariful Islam, Md. Mokbul Hossain

**Affiliations:** 1 Department of Pathology, Faculty of Veterinary Science, Bangladesh Agricultural University, Mymensingh, Bangladesh; 2 Department of Microbiology and Hygiene, Faculty of Veterinary Science, Bangladesh Agricultural University, Mymensingh, Bangladesh

**Keywords:** Salmonellosis, vaccination, shedding, seroconversion, pathology

## Abstract

**Objective:**

Here, we developed and tested the efficacy of a vaccination protocol based on a commercially available live attenuated *Salmonella enterica* serovar Gallinarum (*Salmonella gallinarum*) in layer chickens.

**Materials and Methods:**

50 layer chickens of 16 weeks age were obtained and divided into two groups (*n* = 25), control and vaccinated. The vaccinated group received *Salmonella* vaccine at 0.2 ml/bird, s/c route at 16, 18, 30, and 42 weeks of age. At 21 weeks of age, birds from both groups were challenged with *S. gallinarum* orally at 4 × 10^7^ colony-forming unit per bird.

**Results:**

Both rapid serum plate agglutination and enzyme-linked immunosorbent assay demonstrated a rising rate of seroconversion in vaccinated birds across the study period, with a 4% positive rate at 18 weeks, 56% at 21 weeks, 60% at 30 weeks, and 64% at each time point of 42 and 54 weeks. The vaccine showed 100% clinical protection and reduced the *Salmonella* shedding in the feces and eggs of the challenged birds. On the contrary, the unvaccinated challenged birds showed clinical signs and lesions typical of *Salmonella* infections with morbidity and mortality rates of 36% and 20%, respectively, and had high rates of *Salmonella* shedding in feces and eggs.

**Conclusions:**

With the proposed vaccination schedule *Salmonella* shedding was prevented, and a high seroconversion was confirmed. To prevent *Salmonella* infections in laying flocks, a 3-month interval immunization program is advised starting at the pre-laying stage.

## Introduction

In recent years, Bangladesh has been one of the fastest developing economies in Asia-Pacific. Bangladesh‘s gross domestic product (GDP) is predicted to increase by 5.5% in 2021 and 6.8% in 2022 [1]. Among various sectors contributing significantly to the country’s GDP, poultry farming has developed into a powerful agribusiness during the last two decades. For example, during 2018–2019, the livestock and poultry sector contributed 1.47% to the national GDP [2]. In fact, the country has achieved self-sufficiency in meat and eggs with a production of 76.74 lakh metric tons of meat and 17,360.43 million eggs during 2019–2020 [2]. In the next 5 years, per capita, yearly poultry meat and eggs consumption is predicted to rise by 26% and 41%, respectively [3]. The poultry industry is expected to increase at a similar rate. In fact, by 2024, Bangladesh‘s poultry industry plans to export eggs and poultry meat.

As a result of the rapid growth in poultry industry, the occurrence of diseases has also increased many folds, and the disease continues to be a major problem affecting the sustainability of this industry. Salmonellae is of major economic and public health importance, with poultry and chicken products being one of the most common carriers. Salmonellosis in birds can be caused by one or more members of the *Salmonella* genus, which belongs to the Enterobacteriaceae family. *Salmonella* causes gastroenteritis, typhoid fever, and paratyphoid fever in people and animals [4–6]. Pullorum disease, fowl typhoid, and paratyphoid sickness have grown common in Bangladeshi poultry [7,8]. Most importantly, the fowl typhoid has become a persistent problem in poultry production worldwide [9], causing high hen morbidity and mortality and significant output losses.

Increased biosecurity and vaccination are the primary strategies for preventing illnesses on farms. In developing nations, vaccination has proven to be the most practicable and effective technique for controlling fowl typhoid [10]. A variety of live attenuated vaccinations for hens are available all around the world. Among them, live attenuated vaccines are preferred because they stimulate cell-mediated and humoral immune responses, giving protective immunity [11,12]. Certain live *Salmonella *vaccines have been shown to cross-protect between serotypes [13]. Notably, *Salmonella gallinarum* 9R (SG9R) vaccine strain that protects hens against both *S. gallinarum* and *Salmonella enteritidis* infections has been identified. Importantly, SG9R vaccine strain has been described that protects chickens from both *S*. *gallinarum* and *S*. *enteritidis* infections [14]. However, limited studies have been conducted to develop an effective *S.* *gallinarum* vaccination regimen that can provide clinical protection and prevent cloacal shedding of the bacteria [15].

*Salmonella* vaccine is being imported for commercialization in a few commercial enterprises in Bangladesh. Imported vaccines are used without going through a field study, which should have been required for efficacy evaluation. The immunogenicity of vaccines must be investigated, and a more effective immunization regimen must be recommended.

The fowl typhoid live lyophilized vaccine based on the SG9R strain (9R VAC^R^, Komipharm International Co. Ltd., Korea) is commercially available in Bangladesh. Priming of the 9R VAC^R^ is performed at 6 weeks subcutaneously and then at 12 weeks after priming. However, layer birds are kept for about 100 weeks in Bangladesh, and no standard vaccination protocol for *Salmonella* vaccination is available in the laying stage. The current study looked into designing a cost-effective* Salmonella* vaccination regimen starting at the pre-laying stage with a 3-month interval, allowing farmers to save money while ensuring vaccine efficiency. 

## Materials and Methods

### Ethical approval

The research was carried out in compliance with Bangladesh Agricultural University‘s Ethical Standard of Research Committee in Mymensingh (BAURES 2020 ESRC/VET/07). The Ethical Standard of the Research Committee reviewed and approved the protocol and procedures employed.

### Chickens

A total of 50 layer chickens of Institut de Sélection Animale Brown breed of 16 weeks age were purchased from Diamond Hatchery Ltd. Gazipur, Bangladesh The birds were separated into two groups: unvaccinated challenged (group A, *n* = 25) and vaccinated challenged (group B, *n* = 25), and reared in two separate sheds with adequate biosecurity measures. The birds were given ad libitum access to a commercially prepared diet and fresh drinking water.

### Salmonella isolates and vaccine

For experimental infection, a pure culture of *S. gallinarum* (Isolate No. 4) was propagated in the laboratory and confirmed. The polymerase chain reaction was performed to to confirm the isolate. Commercial fowl typhoid live lyophilized vaccine is used for vaccination (SG-9R Strain, Komipharma, South Korea). Rafiq Medicine Bangladesh (Shimultola, Savar, Dhaka) imports this vaccine for commercial use in poultry of Bangladesh. The vaccine employed in this study was obtained from a local pharmacy.

### Vaccination and challenge infection

At 16 weeks of age, chickens from the vaccinated challenge group (group B) received subcutaneous administration of fowl typhoid live lyophilized vaccine (SG-9R Strain, Komipharma, South Korea), which contains 2 × 10^7^ colony-forming unit (CFU) of bacteria (0.2 ml/bird). Booster vaccinations were given at 18 weeks of age, then every 3 months until the flock was 42 weeks old. Chickens from both the unvaccinated and vaccinated groups were given 0.5 ml of nutrient broth culture containing 4 × 10^7 ^CFU of *S. gallinarum* orally at 21 weeks of age. Clinical signs, morbidity, and mortality were recorded from 21 to 54 weeks of age in both challenge groups (unvaccinated and vaccinated) of birds.

### Sample collection

Cloacal swabs were collected in tetrathionate broth for culture and phenotypic isolation of *Salmonella* spp. at 16, 18, 21, 30, 42, and 54 weeks of age from each bird of both unvaccinated and vaccinated groups. Blood samples were collected for the rapid serum plate agglutination (SPA) test and enzyme-linked immunosorbent assay (ELISA) during the same time points. Centrifugation at 3,000 rpm for 10 minutes, sera were isolated from blood, cleared, and stored at −20°C. Eggshell swabs (*n* = 25) were collected at 30, 42, and 54 weeks of age from both groups of birds and cultured.

## Necropsy and histopathology

*Salmonella*-infected dead birds (*n* = 5) or healthy live birds sacrificed at 54 weeks of age (*n* = 45) were analyzed at the time of necropsy. The presence of gross pathogenic alterations was documented. Bacteriological samples were obtained aseptically in sterile tubes containing buffered peptone water (liver, lung, heart, spleen, cecal tonsil, and blood). Tissues from the liver, lungs, heart, and spleen for histological investigation were collected in 10% neutral buffered formalin. Following the standard method, tissues were processed and stained with regular hematoxylin and eosin stain after adequate fixation [16]. 

## Phenotypic isolation and biochemical characterization of *Salmonella* spp.

All collected swab samples were first cultured in tetrathionate broth (Oxoid, England) and then subcultured into brilliant green agar (BGA; Oxoid, England) and eosin methylene blue (EMB; Oxoid, England) agar, as described previously [17]. The tetrathionate broth (TTB) had turbidity, BGA had a slightly yellowish white colony, Salmonella-Shigella (SS) agar (Biolife, Italy) had a little grey colony, triple sugar iron (TSI) agar (Oxoid, England) had a black colony, nutrient agar had a gray-white colony, EMB agar had a pinkish colony, and MacConkey agar (Techno Pharm, India) had a pale colony. Gram staining revealed Gram-negative bacteria that were rod-shaped, pink, and had short to long chains, as well as single or paired bacteria. To characterize the *Salmonella* isolates and differentiate different species of *Salmonella* spp., biochemical tests such as the five basic sugar fermentation tests [glucose (Wako, Japan), sucrose (Wako, Japan), lactose (Merk, England), mannitol (Beximco Pharma, Germany), and maltose (Techno Pharm, India)], methyl-red test, Voges-Proskauer test, indole test, and dulcitol (Difco, USA) fermentation test were used. The *Salmonella* isolates did not ferment lactose and sucrose, but they did ferment glucose and mannitol with acid and gas generation.

### SPA test

The SPA test was performed using standard *Salmonella* antigen (Nobilis, Holland) manufactured by Intervet International, Holland. On a glass slide, an equal amount (0.02 ml) of *Salmonella* antigen and chicken sera were placed side by side and thoroughly mixed by stirring with a toothpick and rocking. The results were read in just 2 minutes.

### Enzyme-linked immunosorbent assay (ELISA)

The FLOCKSCREENTM *Salmonella* antibody ELISA kit (Guild Hay Ltd., UK) was used to determine the level of anti-*Salmonella* antibodies in the vaccinated flock. At a wavelength of 450 nm, the optical density (OD) values of the sample wells were measured. The mean negative control absorbance was 0.25, and the mean positive control absorbance was 2.5, indicating that the assay was valid.

### Statistical analysis

Graphs were prepared using the statistical software package GraphPad Prism 5.0 software.

## Results

### Fowl typhoid live lyophilized vaccine mounted robust antibody responses in chickens

**Figure 1.**
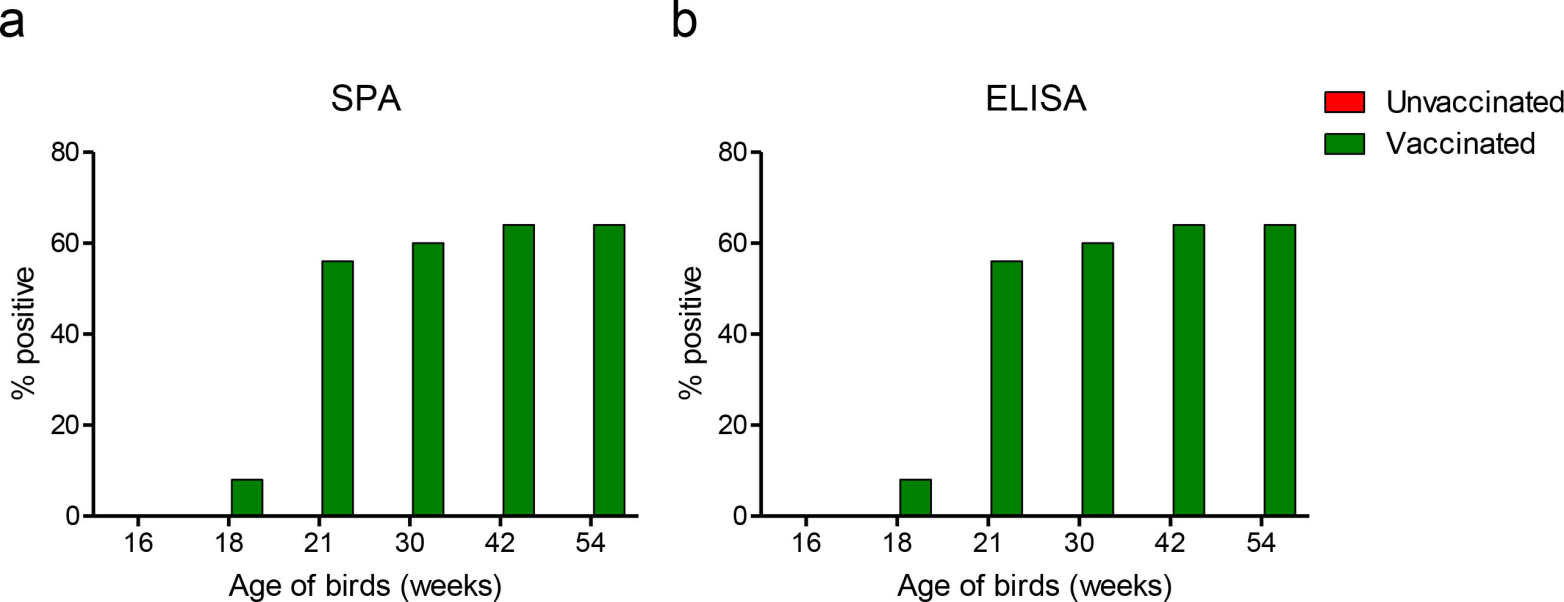
The serum antibody responses in fowl typhoid vaccine-vaccinated chickens. Bar diagram showing the percentage of chickens that tested positive for antibodies in the (a) SPA test and (b) ELISA test.

SPA and ELISA examined antibody responses in hens immunized with fowl typhoid live lyophilized vaccine. At each test, 288 sera samples were collected from vaccinated and unvaccinated chickens at 16, 18, 21, 30, 42, and 54 weeks of age. None of the tested birds showed any detectable antibody response at age 16 weeks (pre-vaccination). While the SPA test demonstrated a rising seroconversion rate in the vaccinated birds over the study period (Fig. 1a), with a 4% positive rate at 18 weeks, 56% at 21 weeks, 60% at 30 weeks, and 64% at each time points of 42 and 54 weeks. Similarly, the antibody response detected by the ELISA also revealed a similar seroconversion as detected by the SPA test (Fig. 1b). Taken together, the fowl typhoid vaccine mounted significant antibody responses in chickens.

### Fowl typhoid live lyophilized vaccine protected chickens upon S. gallinarum challenge

**Figure 2.**
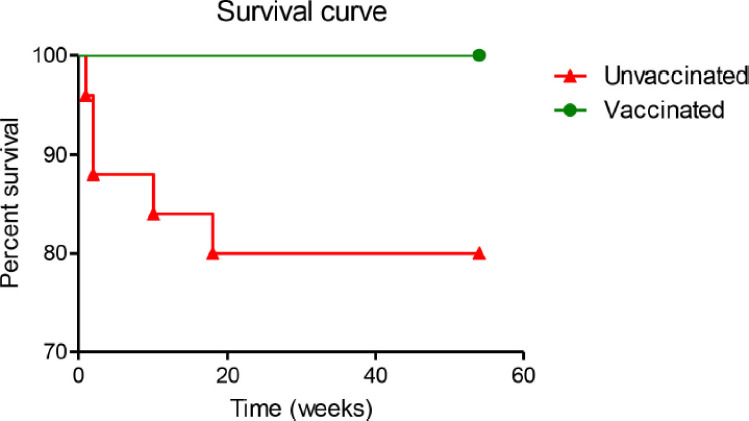
Survival curve of fowl typhoid vaccinated and unvaccinated chickens upon challenge with *S. gallinarum*.

Chickens in both vaccinated and unvaccinated groups were challenged with *S. gallinarum,* and the clinical signs, morbidity, and mortality were recorded. The vaccinated challenged chickens did not show any signs of illness throughout the study period. On the other hand, nine chickens from the unvaccinated challenged group became sick, and five of them died with one bird at each time point of 3, 10, 14, 70, and 126 days post-infection (Fig. 2). The morbidity and mortality of the unvaccinated challenged birds were 36% and 20%, respectively.

### Gross and histopathological changes in Salmonella-infected chickens

**Figure 3.**
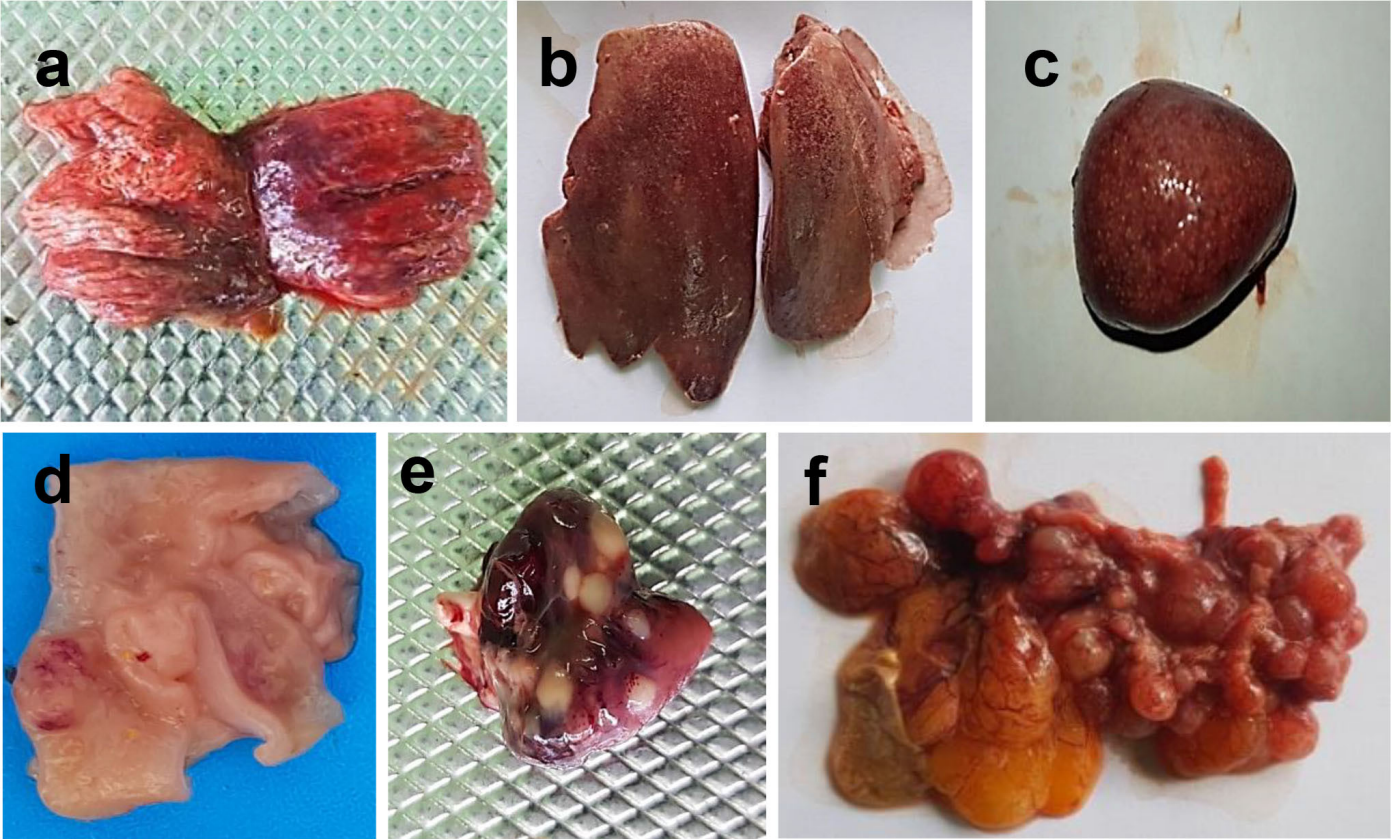
Gross pathological changes in unvaccinated chickens infected with *S. gallinarum*. Hemorrhages and congestions in (a) Lungs and (b) Liver, (c) Hemorrhages, congestion, and necrosis in the spleen, (d) Hemorrhages and swelling of the cecal tonsil, (e) Pericarditis with nodules in the heart, and (f) Hemorrhages, congestion and stalk formation in the egg follicles.

The gross pathological changes *Salmonella* infected chickens from both vaccinated and unvaccinated groups were analyzed. The unvaccinated challenged birds showed much more significant pathological changes than those vaccinated (Fig. 3). The major gross pathological changes of the unvaccinated challenge birds included hemorrhages and congestion in the lungs (84%) and liver (80%) (Fig. 3a and b). Other liver lesions had enlarged fragile and bronze-colored liver (20%), white necrotic foci (44%), and nodule formation (4%). Hemorrhages, congestion, and necrosis in the spleen (40%) (Fig. 3c), kidney (32%), and cecal tonsils (68%) (Fig. 3d) were also observed. Pericarditis with necrotic foci/nodules in the heart (Fig. 3e) was found in 24% of birds. The ovarian lesions included hemorrhages, congestion, and stalk formation in the egg follicles (88%) (Fig. 3f). On the other hand, the vaccinated challenged birds showed hemorrhages and congestion in the lungs (24%), liver (24%), cecal tonsils (16%), and egg follicles (16%).

**Figure 4.**
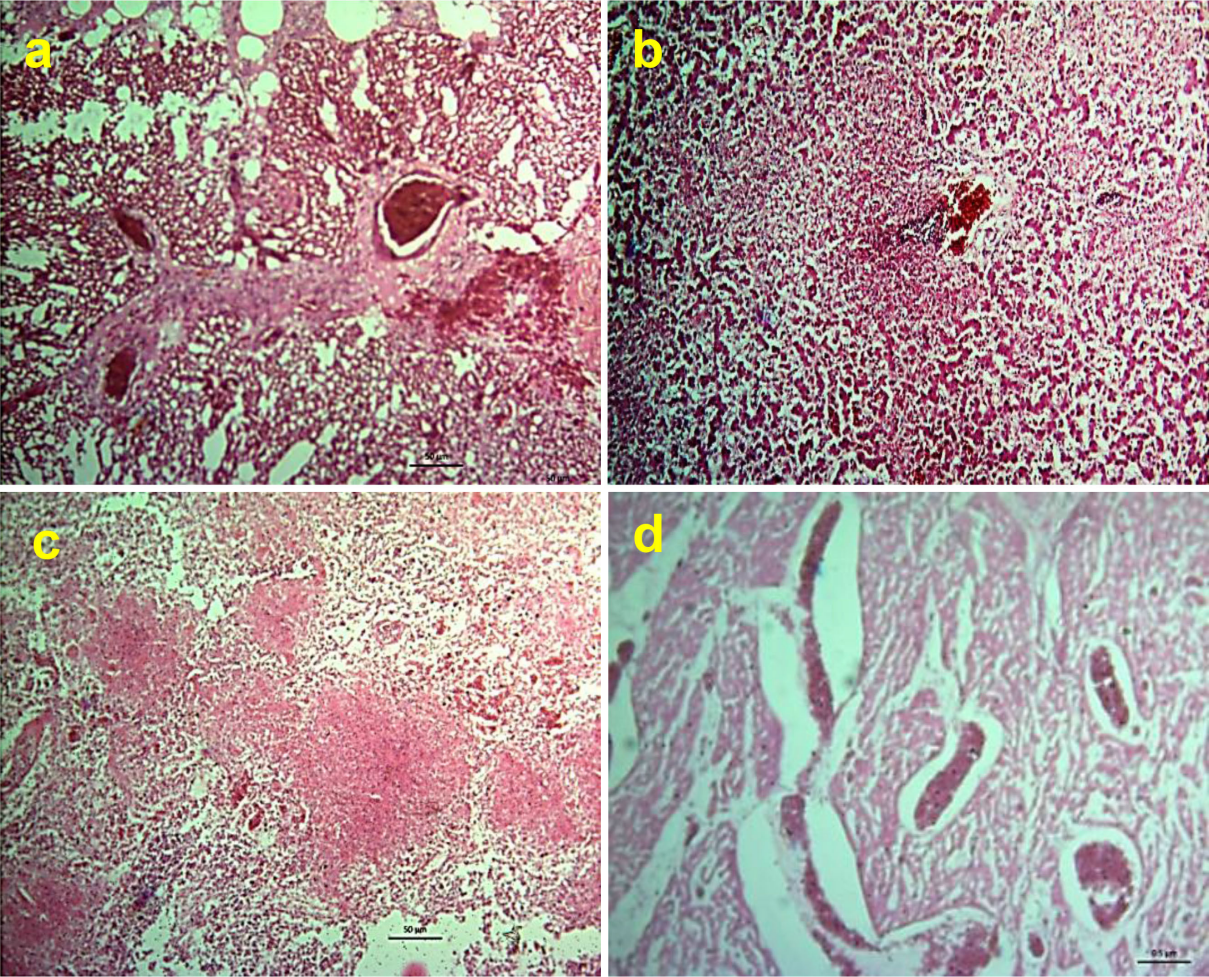
Histopathological changes of unvaccinated chickens challenged with *S. gallinarum*. Section of tissues showing (a) Congestion, hemorrhages, and infiltration of inflammatory cells in the lungs and (b) Liver, (c) Congestion, hemorrhages, and focal necrosis in the spleen, and (d) Congestion in the myocardium of the heart.

Like the gross pathology, most histopathological changes were found in unvaccinated infected chickens. Congestion, hemorrhage, and infiltration of inflammatory cells were found in the lungs of 84% of birds (Fig. 4a). Lesions in the liver included congestion, necrosis, and infiltration of inflammatory cells (80%) (Fig. 4b), focal necrosis with multifocal infiltration of heterophils (36%), and nodular changes with infiltration of macrophages, lymphocytes, plasma cells, and heterophils (4%). Severe congestion and lymphocytic necrosis (Fig. 4c) were found in the spleen of 48% of birds. Heart showed congestion (Fig. 4d), infiltration of heterophils, macrophages, lymphocytes, and plasma cells in the pericardium and myocardium (20%), and nodule formation (4%). Congestion with infiltration of heterophils was found in kidneys (32%). Hemorrhages, congestion, and infiltration of inflammatory cells were found in egg follicles of 88% of birds. But in the vaccinated group, only 24% of birds showed congestion, hepatitis, and infiltration of inflammatory cells in the liver. About 24% of the vaccinated challenged birds showed congestion and hemorrhages in the lungs, while 16% of the birds showed congestion, hemorrhages, and infiltration of inflammatory cells in the egg follicles. The heart, kidneys, and spleen show overall normal histology.

### Fowl typhoid live lyophilized vaccine reduced Salmonella shedding in feces and eggs

**Figure 5.**
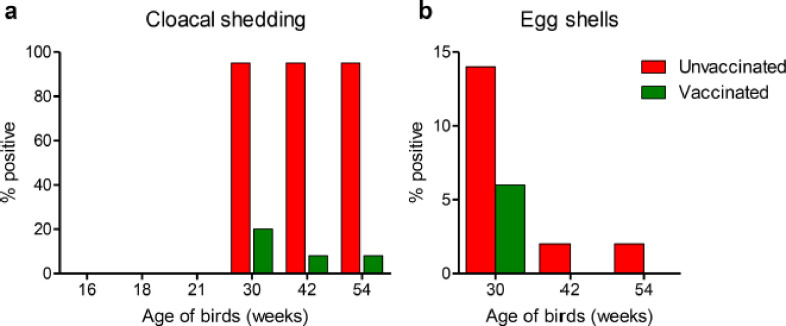
Shedding of *Salmonella* spp. in feces and eggs. Bar diagrams showing the percent of birds shedding *Salmonella* in cloacal swabs (a) and eggshell surfaces (b) from both vaccinated and unvaccinated chickens.

Cloacal swabs were collected from vaccinated and unvaccinated challenged birds, and *Salmonella* spp. were isolated. 95% of the cloacal swabs from the unvaccinated challenged birds carried *Salmonella* spp. at each time point of 30, 42, and 54 weeks. On the contrary, the vaccinated challenged birds had comparatively reduced cloacal shedding with 20%, 8%, and 8% positive rates at 30, 42, and 54 weeks, respectively (Fig. 5a). 

We then tested the presence of *Salmonella* spp. on the eggshell surface. 100 eggshell swabs, 50 from each group, were collected at each time point of 30, 42, and 54 weeks. In unvaccinated chickens, *Salmonella* spp. were detected in 14%, 2%, and 2% of the eggshell swabs at 30, 42, and 54 weeks, respectively (Fig. 5b). On the other hand, only 6% of eggshell swabs contained *Salmonella* spp. at 30 weeks in vaccinated chickens with no *Salmonella* spp. was detected at 42 and 52 weeks (Fig. 5b). Our findings showed decreased *Salmonella* shedding in feces and eggshell after the flock was vaccinated.

## Discussion

Small-scale commercial farms, which usually rear several hundred to a few 1,000 birds with minimum biosecurity measures, are predominating in Bangladesh, and the birds are more vulnerable to Salmonella infections than the large-scale commercial farms. *Salmonella* infections create substantial economic losses along with a reduction in productivity and high mortality [18,19]. The most effective means of controlling *Salmonella* infections in poultry is the result of a mix of strict biosecurity procedures and eradication efforts [20,21]. Vaccination against *S. gallinarum* is intended to improve avian resistance to *Salmonella* infection and reduce feces and egg shedding [12,22]. 

Bangladesh is planning to export poultry meat and eggs shortly. Among the different terms of reference for exports of poultry products, *Salmonella*-free meat and eggs are inevitable. Reviewing the available literature (vaccination at day 1 and boosting at 6, 10, 14 weeks) [23,24] and from our pilot experiments, a protective and cost-effective (in terms of vaccine and labor costs) *Salmonella* vaccination at 3 months interval up to the last stage of laying was designed [25]. This 3-month interval protocol showed desired effects of protection by the challenging dose of *Salmonella,* and it was cost-effective due to the long interval of vaccination [25,26].

In this study, we applied a live attenuated SG9R [27] commercial vaccination in a layer flock at 16 weeks of age, followed by boosting at 18 weeks, and then boosting every 3 months. The immunization schedule elicited robust antibody responses, reduced *Salmonella* shedding in feces and eggs, and avoided clinical illness.

Our results corroborate two previous studies that demonstrated the SG9R vaccine is a safe and effective way to keep *S. gallinarum* infections in laying hens under control [14,28]. A recent study used a live commercially attenuated *S. enteritidis* vaccine based on strain Sm24/Rif12/Ssq (AviPro^®^ Salmonella Vac E, ELANCO) to test the protection of seven different immunization schedules in layers [15]. After three vaccination doses during the laying time, the hens were protected, with less shedding and egg contamination and better protection throughout the laying period [15]. *Salmonella* vaccines can also offer inter-serotypic cross immunity. For example, a study showed that layer chickens are protected against *Salmonella typhimurium*, *S. gallinarum*, and *S. enteritidis* infections when given a live attenuated *Salmonella enterica* serovar *typhimurium* strain at 7 days of age followed by boosting at 5 weeks of age [29].

Similarly, a genetically engineered live attenuated *S.* *enteritidis* vaccine applied at day-old followed by boosting at 5 weeks of age cross protects chickens against *S.* *typhimurium* infection [13]. The live attenuated SG9R vaccine strain has a severe drawback: it can revert to pathogenicity and cause immunosuppression in young chickens [30]. This residual pathogenicity of the SG9R strain was optimally detoxified by the knockout of essential virulence genes and thereby can be used in preventing fowl typhoid irrespective of the age of chickens [20].

About 36% of the unvaccinated infected chickens showed clinical signs and lesions characteristics of fowl typhoid, and 20% of the infected birds died. Variable morbidity and mortality rates in chickens infected with *S.* *gallinarum* have been reported in earlier studies depending on the age and immune status of the birds, virulence of the bacterial strains, management status, rearing system, biosecurity measures, etc. For example, the *S.* *gallinarum* produces very high mortality (40%–100%) in chicks with severe inflammation in the liver and spleen [29,31,32]. However, the mortality rates of *S.* *gallinarum* infection in layer chickens varied greatly between studies. A high mortality rate (95%–100%) was reported in an experimental infection study in layer chickens [33]. Although, a similar study in layers also reported a low (4%) mortality rate [34]. Nevertheless, this study‘s gross and histopathological changes marked by necrotic and hemorrhagic changes in liver, spleen, lungs, and egg follicles corroborate with several previous *S.* *gallinarum* challenge studies [29,31,32].

Cell-mediated immunity (CMI) is critical in the fight against intracellular microorganisms. Humoral immunity, such as antibody-dependent cellular cytotoxicity (ADCC), has a vital role in combating infection. The neutralization of intracellular antigen is not so effective by humoral antibody response; however, it may play some role when antigens present outside the cells [35,36]. In the CMI response investigation, estimation of cytokines (especially IL-2 and IFN-γ) is measured. However, in this experiment, the CMI response was not included. For combating the intracellular infection, both CMI and humoral response are almost equally important [37,38].

The effectiveness of any *Salmonella* vaccination program is determined by the vaccine‘s level of clinical protection and the vaccine‘s ability to prevent *Salmonella *shedding in feces and eggs. One of the most prominent transmission mechanisms of poultry Salmonellae transmission is transovarian infection, which leads to the infection of the eggs and the chicks [39]. Moreover, contamination of eggs with zoonotic Salmonellae such as *S.* *enteritidis* and *S.* *typhimurium* has been recorded and is of great concern for public health and international trade restrictions [25,40]. The vaccine schedule employed in this study significantly reduced the shedding of *Salmonella* spp. in feces and eggs compared to the unvaccinated challenged birds. Eventually, no shedding was recorded in eggs after 30 weeks of age, indicating that the vaccine schedule can be used efficiently in producing *Salmonella*-free eggs in the field. 

## Conclusion

In conclusion, the vaccine efficacy test of fowl typhoid 9R strain vaccine this study found a high level of seroconversion and that *Salmonella* shedding in feces and eggs was reduced. To protect chickens from *Salmonella* infections and egg contamination, a 3-month interval immunization program starting at prelaying should be followed. Further study should focus on evaluating cell-mediated immune responses in the vaccinated birds to elucidate the mechanism of clinical protection induced by the live lyophilized *S.* *gallinarum* vaccine.

## List of Abbreviations

Colony-forming unit (CFU), Gross domestic product (GDP), Enzyme-linked immunosorbent assay (ELISA), Polymerase chain reaction (PCR), Rapid serum plate agglutination (SPA).
